# Glycogen Synthase Isoforms in *Synechocystis* sp. PCC6803: Identification of Different Roles to Produce Glycogen by Targeted Mutagenesis

**DOI:** 10.1371/journal.pone.0091524

**Published:** 2014-03-17

**Authors:** Sang-Ho Yoo, Byung-Hoo Lee, Youyoun Moon, Martin H. Spalding, Jay-lin Jane

**Affiliations:** 1 Department of Food Science & Technology and Carbohydrate Bioproduct Research Center, Sejong University, Seoul, Korea; 2 Whistler Center for Carbohydrate Research and Department of Food Science, Purdue University, West Lafayette, Indiana, United States of America; 3 Department of Botany, Iowa State University, Ames, Iowa, United States of America; 4 Department of Food Science and Human Nutrition, Iowa State University, Ames, Iowa, United States of America; Consejo Superior de Investigaciones Cientificas, Spain

## Abstract

*Synechocystis* sp. PCC6803 belongs to cyanobacteria which carry out photosynthesis and has recently become of interest due to the evolutionary link between bacteria and plant species. Similar to other bacteria, the primary carbohydrate storage source of *Synechocystis* sp. PCC6803 is glycogen. While most bacteria are not known to have any isoforms of glycogen synthase, analysis of the genomic DNA sequence of *Synechocystis* sp. PCC6803 predicts that this strain encodes two isoforms of glycogen synthase (GS) for synthesizing glycogen structure. To examine the functions of the putative GS genes, each gene (*sll*1393 or *sll*0945) was disrupted by double cross-over homologous recombination. Zymogram analysis of the two GS disruption mutants allowed the identification of a protein band corresponding to each GS isoform. Results showed that two GS isoforms (GSI and GSII) are present in *Synechocystis* sp. PCC6803, and both are involved in glycogen biosynthesis with different elongation properties: GSI is processive and GSII is distributive. Total GS activities in the mutant strains were not affected and were compensated by the remaining isoform. Analysis of the branch-structure of glycogen revealed that the *sll*1393^−^ mutant (GSI^−^) produced glycogen containing more intermediate-length chains (DP 8–18) at the expense of shorter and longer chains compared with the wild-type strain. The *sll*0945^−^ mutant (GSII^−^) produced glycogen similar to the wild-type, with only a slightly higher proportion of short chains (DP 4–11). The current study suggests that GS isoforms in *Synechocystis* sp. PCC6803 have different elongation specificities in the biosynthesis of glycogen, combined with ADP-glucose pyrophosphorylase and glycogen branching enzyme.

## Introduction

Glycogen is the major form of carbon storage in many prokaryotes. It is an α-1,4 linked d-glucose polymer with α-1,6 branches similar to amylopectin, which is a part of starch components in plants with amylose. Biosynthesis of bacterial glycogen requires three enzymes: ADP-glucose pyrophosphorylase (AGPase, EC 2.7.7.27), glycogen branching enzyme (GBE, EC 2.4.1.18), and glycogen synthase (GS, EC 2.4.1.21). These three enzymes have specific individual roles in producing a bacterial glycogen structure. For example, GS has an elongation property, extending an α-1,4 glucose moiety with ADP-glucose as a substrate [1], which is produced by the AGPase reaction. The biosynthetic pathway of bacterial glycogen is very similar to that of starch biosynthesis in plants, using ADP-glucose as a major substrate for starch biosynthesis with different elongation properties for glucose extensions [2], [3]. However, there is an essential difference between bacterial and plant systems regarding whether isoforms of glycogen/starch-synthesizing enzymes are present. Multiple isoforms of starch synthase (SS) and starch branching enzyme (SBE), which have similar enzymatic properties to GS and GBE, have been identified in plants [4] as well as fungi [5]. No isoforms of GS and GBE have been known to exist in bacteria, however, a recent study showed there are two types of glycogen synthases, named as *glg*A1 and *glg*A2, in *Sinorhizobium meliloti* [6].

Cyanobacteria, blue-green algae, are photosynthetic prokaryotes. They contain chlorophyll *a*, the same photosynthetic pigment that plants contain. From an evolutionary point of view, cyanobacteria are considered a linking organism between bacteria and green plants, because they are proposed to represent the endosymbiotic progenitors of plastids in plants [7]. While cyanobacteria have highly organized internal thylakoid membranes similar to those in plant chloroplasts [8], they produce glycogen instead of starch. As in plants and/or other bacteria, cyanobacteria utilizes ADP-glucose as a precursor for the biosynthesis of glycogen instead of UDP-glucose [9]. Therefore, the process of storing carbohydrates through photosynthesis in cyanobacteria may bear a closer resemblance to that in plants than do other bacteria or non-photosynthetic eukaryotes.

It is widely accepted that different specificities of SS and SBE isoforms play important roles in determining starch structure in the plant system [10], [11]. Transposon-tagging and antisense down-regulation of specific genes involved in starch biosynthesis have helped to elucidate different functions for starch synthesizing enzymes [12], [13], but the specific function and metabolic regulation of each isoform in starch biosynthesis are still not fully understood. One of the major difficulties in revealing the starch biosynthetic mechanism by mutation or down-regulation of specific enzyme isoforms is the compensating effect of the remaining isoforms and/or the pleiotropic effect of the mutations on other starch biosynthetic enzymes in plants [14], [15]. Disruption of one SS isoform in potato has been shown not to affect the overall starch yield [16]. A cyanobacterial system may be ideal for indirectly examining the unique functionality of each plant SS isoform, since the cyanobacterium *Synechocystis* sp. PCC6803 appears to have two GS isoforms [17]. These two putative GS genes (GSI and GSII) are expected to have different elongation properties in glycogen biosynthesis like SS isoforms, and would be of use to investigate the starch synthesizing process in plant systems.

In this study, we characterized different functions of two endogenous GS isoforms in the process of glycogen biosynthesis in *Synechocystis* sp. PCC6803. Our ultimate goal is to establish a model system for glycogen biosynthesis in cyanobacteria and then use the model to study the roles of individual enzymes involved in starch/glycogen production. We expect that our findings will provide further information on the evolutionary similarity of α-glucan synthesis between cyanobacteria and plant.

## Materials and Methods

### Materials

ADP[U-^14^C]glucose was purchased from GE Healthcare Life Sciences (Piscataway, NJ). Chemicals for BG-11 medium were purchased from Fisher Scientific (Pittsburgh, PA). Molecular biology reagents were purchased from Promega Biotech (Madison, WI), and other chemicals and antibiotics were from Sigma-Aldrich Co. (St. Louis, MO).

### Strains and Growth Conditions

*Synechocystis* sp. PCC6803 was grown at 25°C under continuous illumination with fluorescent lights (∼40 µmoles photons m^−2^ s^−1^) in a BG-11 medium supplemented with 5 mM glucose to increase growth rate [18]. For different mutants, media contained 50 µg mL^−1^ kanamycin and/or 20 µg mL^−1^ spectinomycin. For glycogen isolation, cells were grown in a BG-11/glucose medium for 7–8 days (A_730_ = 1.6–1.8) before being transferred to a nitrogen-limiting BG-11/glucose medium, and then grown for another 2–3 days. The nitrogen-limiting BG-11 medium contained 8.34 mg mL^−1^ sodium nitrate as the only nitrogen source, which supplied less nitrogen concentration than 10% of the normal sodium nitrate concentration. Cell pellets grown in normal BG-11 medium, harvested by centrifugation at 6,000× *g* for 15 min, were washed with sterile deionized water prior to transfer of cells to a nitrogen-limiting medium. Cultures were started with an initial cell density of 1.0×10^6^ cells mL^−1^ for determination of the cell growth rate.

### Genomic DNA Isolation

For isolation of genomic DNA from *Synechocystis* sp. PCC6803 cell cultures in early stationary phase, cell pellets collected by centrifugation of 5 mL samples were resuspended in 400 µL TE buffer [50 mM Tris-HCl, pH 8.0, and 5 mM ethylenediaminetetraacetic acid (EDTA)] following a general protocol reported by Ausubel et al. [19]. The same volume (400 µL) of glass beads (acid washed, baked) was added to the cell suspension, followed by the addition of SDS (10%, w/v, 8 µL), *N*-lauryl sarcosine (5%, w/v, 16 µL), and phenol saturated with TE (400 µL). The cell suspension was then vortexed five times, 15 s each.

### Targeted Mutagenesis of *sll*1393 and *sll*0945 Genes

Mutants lacking putative GS genes, *sll*1393 or *sll*0945, were generated by transforming the wild-type strain of *Synechocystis* sp. PCC 6803 with pSHK1393 or pSHK0945 plasmid, respectively, in which *sll*1393 or *sll*0945 was replaced by a gene conferring kanamycin resistance. DNA sequences from both 5′ and 3′ regions of each targeted structural gene were used to amplify corresponding ∼0.5 kb fragments. Primers were obtained from *sll*1393 and *sll*0945 genes to avoid possible mutagenesis in flanking regions during recombination events (Table 1). Two sets of primers, prA1 (forward)/prA2 (reverse) and prA3/prA4 for *sll*1393 were used for PCR amplification. For *sll*0945, prB1 (forward)/prB2 (reverse) and prB3/prB4 were used. Primers were designed to contain restriction sites at both ends of the amplified DNA fragments as shown in Table 1. Endonuclease-restricted *Bam*HI-*Eco*RI and *Eco*RI-*Hind*III fragments from each gene were subcloned sequentially into pBluescript KS vector. A kanamycin resistance (Km^R^) gene from pUC4K was inserted between subcloned DNA fragments using the *Eco*RI restriction site. Generated mutants in which either *sll*1393 or *sll*0945 was disrupted were named M1 and M2, respectively. The knock-out plasmid pSHS1393 was constructed to target disruption of the *sll*1393 gene by ligating an *Eco*RI-fragment of the spectinomycin resistance (Sp^R^) gene from pHP45Ω [20] into pSHK1393 instead of the Km^R^ gene. Attempts to generate a double mutant (M12 or M21), in which both *sll*1393 and *sll*0945 genes were disrupted, were performed by transforming a pSHK0945-transformed mutant (M2) with pSHS1393 or by transforming a pSHK1393-transformed mutant (M1) with M2 genomic DNA. The selection was carried out by using glucose in the media, low CO_2_ without glucose, or high CO_2_ without glucose under antibiotic pressure. After transformants resistant to kanamycin and/or spectinomycin were segregated by single colony selection for several rounds, genomic DNA was isolated. Insertion of the Km^R^ or Sp^R^ gene cassette into the targeted sequence was confirmed by PCR amplification analysis. Growth of wild-type and mutant strains was monitored by measuring absorbance of cell cultures at 730 nm.

**Table 1:** Oligonucleotide sequences used to construct 3 pSHK1393 and pSHK0945 plasmids.

	Oligonucleotides^a^	Position	Site inserted
*sll1393* (1476 bp)			
prA1	5′-GTCGAGCGGATCCTACCCATGT-3′	109–130 (22 bp)	BamHI
prA2	5′-GATCGTAGCTGAATTCATAACTGTC-3′	559–583 (25 bp)	EcoRI
prA3	5′-CCGCCACCGAATTCAATCTGAG-3′	1009–1031 (23 bp)	EcoRI
prA4	5′-TGTATTCGTAAGCTTCCACATATTG-3′	1441–1465 (25 bp)	HindIII
*sll0945* (1434 bp)			
prB1	5′-TAAAGTTCTGGATCCGTTGGGC-3′	81–102 (22 bp)	BamHI
prB2	5′-AATACCGACTGAATTCCCACTC-3′	651–672 (22 bp)	EcoRI
prB3	5′-GTCCTACACCGAATTCCAGTTA-3′	894–915 (22 bp)	EcoRI
prB4	5′-TTCCATGAAGCTTAATTCCTCCG-3′	1374–1396 (23 bp)	HindIII

a Final constructs were used to replace endogenous *sll*1393 and *sll*0945 genes, respectively, by homologous recombination.

The position of the oligonucleotides in the nucleotide sequences of the *sll*1393 and *sll*0945 structural DNA are indicated. The restriction sites introduced by PCR amplification are underlined.

### Zymogram Analysis for Detecting GS Activity

Cells were harvested during the early stationary growth phase from *Synechocystis* sp. PCC6803 culture (100 mL), collected by centrifugation and resuspended in 1.5 mL of 10 mM Tris-HCl (pH 7.0) containing 1 mM EDTA, 1 mM dithiothreitol (DTT), 0.2 mM phenylmethyl sulfonylfluoride (PMSF), and 1 mM benzamidine. An equal volume of 100–150 µm glass beads was added, and cells were broken with a Mini-beadbeater (Biospec Products, Bartlesville, OK). The method used for zymogram analysis was modified from the procedure of Tyynelä and Schulman [21]. The cell-free extract, obtained as the supernatant of a 15 min, 10,000× *g* centrifugation, was used for detecting activity of GS proteins on a native gel following electrophoresis. The cell-free extract (15–25 µL, containing 10 µg of total soluble protein) was mixed with the same volume of sample loading buffer that contains 50% (v/v) glycerol, 0.25% (w/v) bromophenol blue, and 5 mM DTT in 75 mM Tris-HCl buffer (pH 6.8). Protein concentrations were determined using a protein assay kit (Bio-Rad, CA, USA) with bovine serum albumin (BSA) as a standard. Native gel electrophoresis was carried out at 70 V using 7.5% polyacrylamide gel. Electrophoresis finished within 2 h. The gel was removed, gently washed with distilled water, and soaked in 25 mL of 50 mM sodium citrate buffer (pH 7.0) containing 20 mg glycogen and 15 mg ADP-glucose as substrates for *in vitro* synthesis of glucan. The reaction mixture was incubated at 25°C for 16 h, and then the gel was stained with iodine solution (I_2_/KI = 0.01/0.1%, w/v) in 0.1 M sodium acetate buffer (pH 5.0) to detect dark purple-colored protein bands corresponding to GS activity.

### Glycogen Synthase Assay

The assay for the quantitative determination of GS activity was modified from Thomas et al. [22]. A cell-free extract was prepared as described above. Enzyme activity was determined in 50 mM sodium citrate (pH 7.0) containing 10.0 mg glycogen mL^−1^ and 0.5 µmol of ADP[U-^14^C]glucose at 0.08 Ci/mol (3.0 GBq/mol) and 20 µL of the cell extract in a final volume of 220 µL. The reaction was initiated by adding cell-free extract at 25°C. Zero-time control was prepared within 5 sec of the addition of cell extract to the assay mixture. An aliquot (30 µL) of sample was taken from the reaction mixture and spotted immediately on a square (1.5 cm×1.5 cm) of Whatman No. 3 filter paper at each time interval. Each spotted filter paper was dropped into a beaker containing 50 mL of 75% ethanol (v/v) and was washed twice with an equal volume of 75% ethanol. After being air-dried, the ethanol-insoluble ^14^C retained on the filter paper was determined by liquid scintillation spectroscopy (Model LS 6500, Beckman, Fullerton, CA).

### Glycogen Branching Enzyme Assay

GBE activity was determined using a phosphorylase stimulation assay [23]. The enzyme extract (as above) was centrifuged at 50,000× *g* for 1 h at 4°C, and 4 µL of the supernatant diluted in 86 µL of deionized water was combined with an assay solution (20 µL of 10 mM AMP, 20 µL of 1 mg/mL phosphorylase *a*, and 20 µL 1 M sodium citrate, pH 7.0, in a final volume of 85 µL), and the reaction was initiated by addition of 25 µL of 400 mM glucose-1-phosphate (G1P) and then incubated at 30°C. At 0.5, 5, 10, 30, 60, and 90 min, 25 µL samples were removed, diluted with 175 µL of deionized water and boiled for 10 min to stop the reaction. The phosphate released was determined using Malachite-Green assay [24].

### ADP-Glucose Pyrophosphorylase (AGPase) Assay

The assay for AGPase activity measured the phosphate (P_i_) released by the combined actions of AGPase and inorganic pyrophosphatase, modified from Steiner and Preiss [25]. The reaction was initiated by combining 450 µL of a reaction premix (200 mM HEPES, 1 mg/mL BSA, 3 mM ATP, 10 mM MgCl_2_, 20 mM G1P and 1.3 units/mL inorganic pyrophosphatase, pH 7.0) with an enzyme solution (135 µL extract and 315 µL of water), both pre-incubated to a temperature of 37°C. The reaction proceeded at 37°C, and 100 µL samples were withdrawn at 20 sec, 40 sec, 60 sec, 2 min, 3 min, 4 min and 5 min. Each reaction was terminated in a boiling water bath. The inorganic phosphate content in each sample was determined using Malachite-Green assay [24].

### Determination of Glycogen Yield and λ_max_ of Iodine-Glycogen Complex

Wild type (WT) and mutant cell cultures (1 L; grown for 2 days in *N*-limited BG-11 medium as described above) were harvested at 6,000× *g* for 15 min. The cell pellets were ground in liquid nitrogen using a pestle and mortar, and the soluble glucan was extracted by suspension of the broken cells in 10 mL deionized water and washed twice with an equal volume of water. After centrifugation (10,000× *g* for 15 min), the supernatant was collected and boiled for 15 min. The production yields of glycogen were determined using an enzymatic assay [26] after complete hydrolysis with 5 units mg^−1^_total carbohydrate_ of amyloglucosidase as described previously by Yoo et al. [18]. Total carbohydrate content was estimated following the phenol-sulfuric acid method [27]. Iodine staining of the glycogen dispersion (0.5 mg mL^−1^) was prepared in a solution containing 0.01% I_2_ and 0.1% KI. Absorbance of the iodine complex was scanned from 700 to 400 nm to determine the wavelength of maximum absorbance (λ_max_).

### Preparation of β-Limit Dextrins of Glycogens and Starch

Cyanobacterial glycogens, rabbit-liver glycogen (Sigma-Aldrich Co., St. Louis, MO) and waxy maize starch (Cargill, Minneapolis, MN) were used for production of β-limit dextrins. Samples (50 mg) were pre-wetted with water (0.5 mL), and then 4.5 mL of DMSO was added. The resulting mixtures were heated in a boiling water bath for 1 h and were precipitated with 5 volumes of absolute ethanol. The air-dried pellets were dissolved in 50 mM acetate buffer (pH 4.8), and boiled for another 0.5 h. After cooling down the dispersions to ambient temperature, sweet potato β-amylase (10 units mg^−1^ glucan, Sigma-Aldrich Co., St. Louis, MO) was added. The hydrolysis reaction proceeded at 37°C for 24 h, and then another 10 unit mg^−1^ of β-amylase was added to completely hydrolyze the external α-1,4 linkages for 24 h. The resulting samples were precipitated with 2 volumes of ethanol, washed twice with 75% (v/v) ethanol, and then once with 100% ethanol. The air-dried pellets, β-limit dextrins, were used for further analysis.

### Analysis of Glycogen and β-Limit Dextrin Structures

To examine the branch structure of glycogen produced by *Synechocystis* sp. PCC6803, 20 mg of glycogen were dissolved in 5 mL of 10 mM acetate buffer (pH 3.5), and 5 µL (300 units) of isoamylase from *Pseudomonas amyloderamosa* (Hayashibara Biochem Lab., Japan) were added to hydrolyze α-1,6 linkages. The reaction mixture was incubated for 48 h in a shaking water bath at 40°C with 120 strokes/min. β-Limit dextrins were dissolved with 30 min boiling to the concentration of 1 mg mL^−1^ in 10 mM acetate buffer (pH 3.5), and incubated with isoamylase (10 units mg^−1^) at 40°C for 24 h. The pH of resulting samples was adjusted to 5.0, and samples were further hydrolyzed with *Klebsiella pneumoniae* pullulanase (10 units mg^−1^, Sigma-Aldrich Co., St. Louis, MO) at 40°C for 24 h. To prevent microbial growth during the enzyme reaction, 10 µL of 10% (w/v) sodium azide solution was also added. Upon completion of the enzyme reaction, the pH was adjusted to 6.0 and the solution was heated for 15 min in a boiling water bath. The branch chain length distributions of glycogen and β-limit dextrins were determined by separation of the hydrolyzed branches using an HPAEC system (Dionex-300, Sunnyvale, CA) equipped with an amyloglucosidase reactor and a pulsed-amperometric detector (PAD) [28]. A PA-100 anion exchange analytical column (250×4 mm) with a guard column was used for separating 25 µL samples of debranched samples. Eluent A and B consisted of 100 mM NaOH and 100 mM NaOH containing 300 mM NaNO_3_, respectively. The gradient of eluent B at 0, 39, 50, 170, and 220 min was 1, 5, 8, 30, and 45%, respectively, with an operating flow rate at 0.5 mL min^−1^.

## Results and Discussion

As *Synechocystis* sp. PCC6803 is considered an evolutionary connector between green plants and bacteria [29], it is expected that there would be many similarities between these species in α-glucan production. The presence of two GS isoforms in *Synechocystis* sp. PCC6803 was identified with differently expressed proteins from mutants (M1 and M2). These findings suggested that each GS of *Synechocystis* sp. PCC6803 might have different enzymatic roles in glycogen biosynthesis.

### Mutagenesis of Putative Glycogen Synthase Genes *sll*1393 and *sll*0945 in *Synechocystis* PCC6803

**Figure 1:**
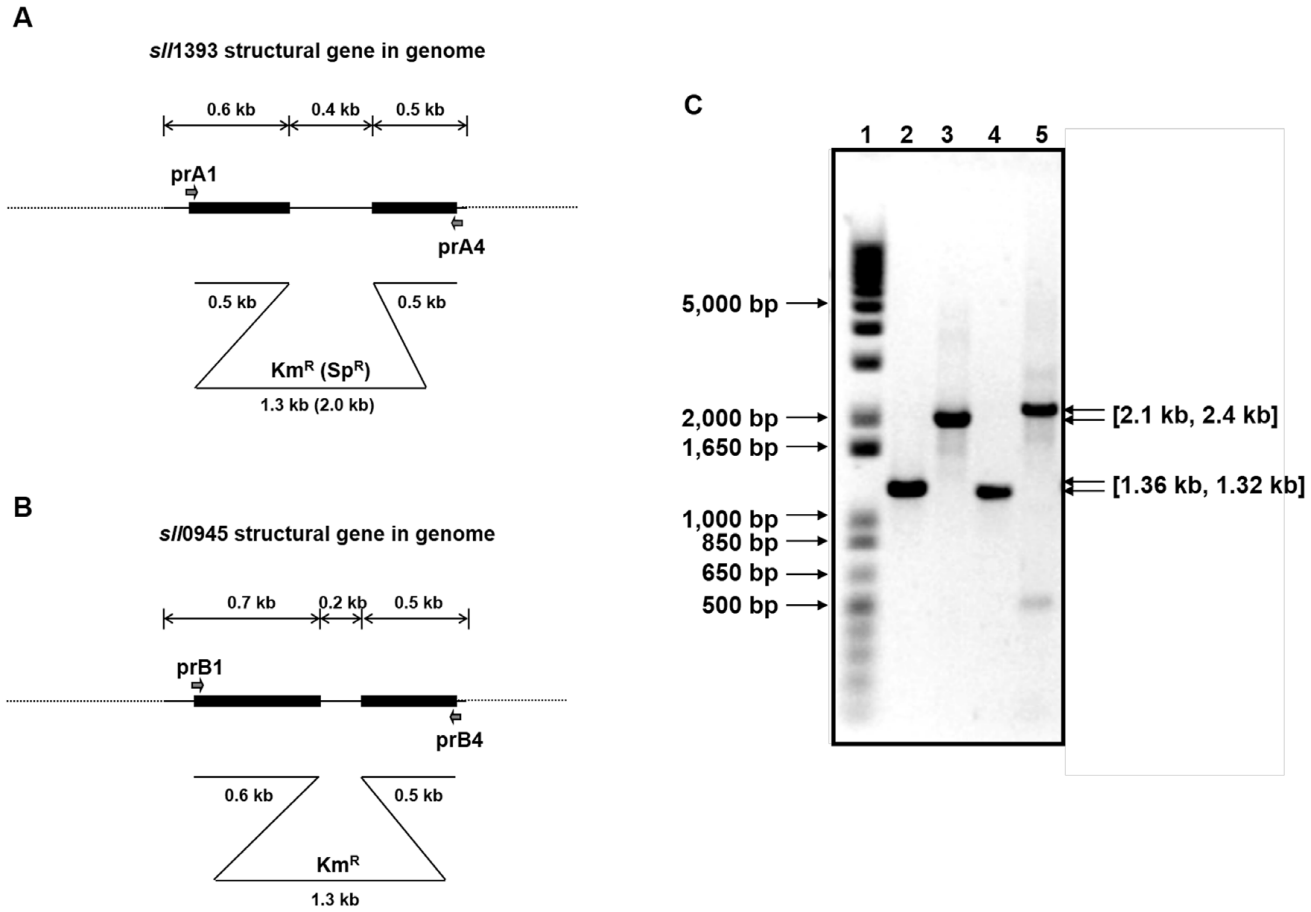
Gene structure of *sll*1393 (A) and *sll*0945 (B) on *Synechocystis* sp. PCC 6803 genomic DNA. The targeted replacement of each GS gene with the Km^R^ gene was achieved by homologous recombination. (C) PCR analysis of *sll*1393 and *sll*0945 genes using genomic DNA from wild-type, *sll*1393^−^, and *sll*0945^−^ strains as templates. For comparison, PCR products of intact genes in wild-type were loaded next (left lane) to the corresponding mutated genes. Lane1 is the size marker (1 kb plus DNA ladder, Life Technologies). DNA bands on lane 2 (WT) and 3 (M1) were amplified using prA1 and prA4; Lane 4 (WT) and 5 (M2) were amplified using prB1 and prB4.

**Figure 2:**
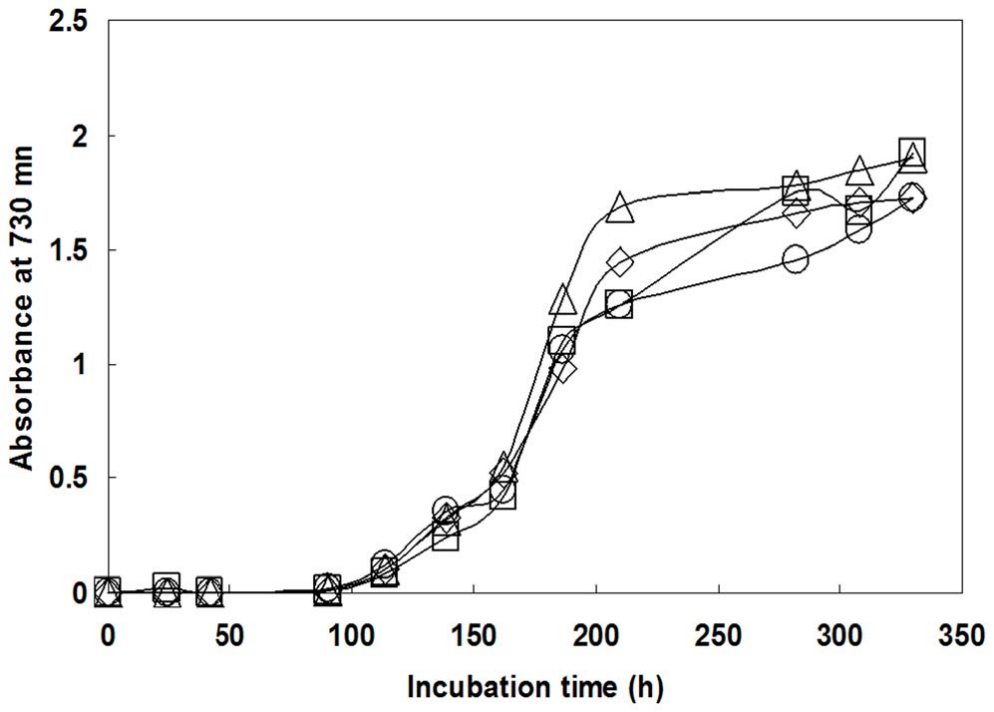
Photomixotrophic growth of WT (▵), M1 (□), M2 (○), and M12 (⋄) strains of *Synechocystis* sp. PCC6803. Cells were grown in a BG-11 medium containing 5 mM glucose and the number of cells were counted in the exponential phase. The same amount of the cells were inoculated in the new media. Each culture was inoculated with an equal number of exponential-phase cells, and growth of the cultures was monitored by measuring absorbance at 730 nm at various time intervals.

The *sll*1393 and *sll*0945 in genomic DNA of *Synechocystis* sp. PCC6803 were inactivated by double cross-over homologous recombination to determine whether or not both genes are encoded functional GS proteins. Structures of *sll*1393 and *sll*0945 are shown in Figure 1A and 1B, respectively. Since *Synechocystis* sp. PCC6803 contains multiple genome copies, complete segregation is necessary prior to functional analysis [30]. Therefore, two single mutants, M1 and M2, were generated by natural transformation of wild-type *Synechocystis* sp. PCC6803 with plasmids pSHK1393 and pSHK0945, respectively, both conferring kanamycin resistance. As shown in Figure 1C, in mutant M1 the disrupted *sll*1393 PCR product (2.1 kb) was detected, while there was no PCR product corresponding to the size of intact gene (1.36 kb). Similarly, PCR products from the mutant M2 clearly showed the disrupted *sll*0945 (2.4 kb) but no intact gene (1.32 kb). The actual PCR product size of Km^R^-harboring *sll*1393gene in M1 was different by ∼200 bp from the theoretical estimation depicted in Figure 1A, but there was no reasonable explanation about this discrepancy. The growth patterns were found to be very similar (Figure 2), although M2 was observed to have a slightly reduced growth rate and final culture density. M1 and M2 mutants are expected to have different DNA to produce putative glycogens [31], [32]. Nevertheless, the growth pattern data revealed that the single mutation of GS gene did not have many effects on *Synechocystis* sp. PCC6803 physiology, which corresponds with previous research by Gründel et al. [32].

The double mutation, M12 or M21, was used to test other possible GS activities by sequential transformation using plasmids pSHK0945 and pSHS1393, in either order, conferring kanamycin and spectinomycin resistance, respectively. However, complete segregation of the double mutant was never obtained, regardless of the order of the gene disruptions or whether selection was performed in different growth conditions as described in the methods section. These results suggest that *Synechocystis* requires at least one active GS enzyme in order to survive. Although the reason for this is unclear, one can speculate that synthesis of α-glucans may be essential for glucose homeostasis [32] rather than as a carbohydrate reserve, since the cyanobacteria were grown in constant light and should not need carbohydrate reserves. However, the inability to eliminate both GS genes serves as clear evidence for the lack of any additional GS genes in *Synechocystis*. Moreover, the introduction and expression of a potato starch synthase III gene into *Synechocystis* [33] allowed the complete elimination of both GSI and GSII, which supports the conclusion that *Synechocystis* sp. PCC6803 requires a glucan synthase for survival and that GSI and GSII are the only GS isoforms.

Although attempts at making a double mutant were not successful, insertion of the only Sp^R^ gene without kanamycin resistance gene into mutant M1, and selection on spectinomycin for generating M12 resulted in substantial suppression of GS activity (Table 2). Growth of this nominal “M12” mutant on both antibiotics reduced the growth rate considerably but allowed some characterization of the cyanobacterium with very low GS activity.

**Table 2:** Effects of insertional mutation of *sll*1393 and *sll*0945 genes on GS, GBE, and AGPase activities.

Strain	GS activity^a^ µmol min^−1^ mg^−1^ protein	GBE activity^b^ µmol min^−1^ mg^−1^ protein	AGPase activity^c^ µmol min^−1^ mg^−1^ protein
WT	0.080±0.001	0.07±0.01	0.007±0.001
M1	0.08±0.01	0.07±0.02	0.005±0.001
M2	0.100±0.001	0.12±0.02	0.007±0.002
“M12”	0.018±0.006	ND^d^	ND

a Incorporation rate of [^14^C]glucose onto rabbit-liver glycogen primers.

Values given are means ±standard deviation obtained from three independent experiments.

b Stimulation of glucose incorporation rate.

Values given are means ± standard deviation obtained from two independent experiments with two replicates in each.

c Rate of ADP-glucose formation.

Values given are means ± standard deviation obtained from two independent experiments with a total of four replicates.

d not detected.

### Zymogram Analysis Confirmed Two Detectable Protein Bands with GS Activity

**Figure 3:**
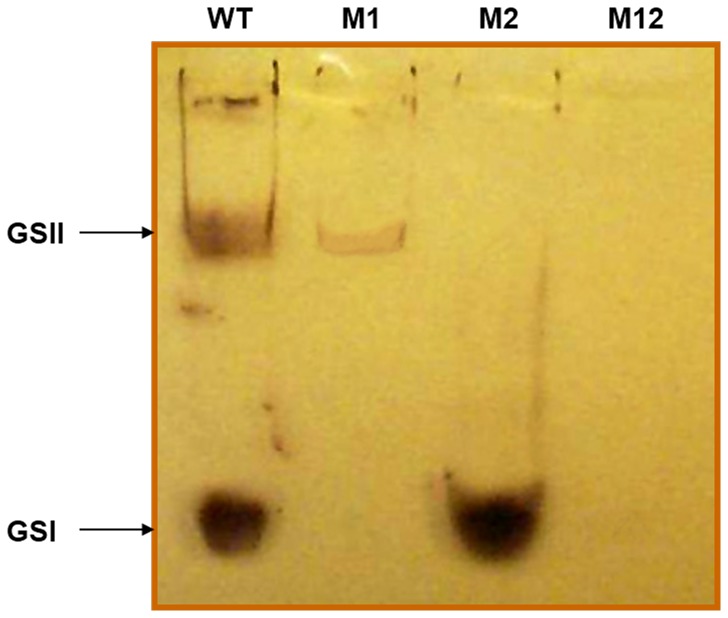
Zymogram analysis of GS activity from wild-type and mutant cells. Ten µg of each soluble extract was subjected to native PAGE. The bands showing GS activity were visible after overnight incubation in a buffer containing ADP-glucose and rabbit-liver glycogen and were stained with iodine solution. Lane 1. WT, Lane2. M1 (*sll*1393^−^), Lane3. M2 (*sll*0945^−^), and Lane 4. M12 (*sll*1393^−^/*sll*0945^−^).

Two different protein bands with GS activity were identified using a native gel zymogram analysis, which is a detecting technique for enzyme activities based on the PAGE technique. Differential color intensity was observed between GSI and GSII in the zymogram for WT, M1 and M2. A strong purple-colored major band (GSI) stained with iodine was detected in wild-type (WT) and was the only detectable band in M2. A minor band (GSII) was also identified in WT and was the only visible band in M1 (Figure 3). These results indicate that there are two distinct GS proteins in *Synechocystis* sp. PCC6803, and the *sll*1393 and *sll*0945 genes encoded GSI and GSII, respectively. The difference in staining intensity and color of the two GS is an indication of a difference in the structures of the rabbit liver glycogen-primed products produced by individual GS enzymes. Longer chains were more intensely stained by I_2_/KI solutions than shorter chains suggesting that the action of GSI (dark purple band) may produce longer α-glucosyl chains than the action of GSII (light brown band) [34]. It is worth noting that there were no apparent detectable GS activity bands in the nominal double mutant, “M12” under these assay conditions.

### Total GS Activities in the Mutant Strains were not Affected and were Compensated by the Remaining Isoform

To examine the total GS activity in WT and mutant strains, the rate of [^14^C]glucose incorporation from ADP[U-^14^C]glucose onto rabbit-liver glycogen was determined. The incorporation rate was fairly linear for all strains over the initial 40 min of the assay. The rates of [^14^C]glucose incorporation for WT, M1, M2, and M12 were 0.08, 0.082, 0.1, and 0.018 µmol glucose min^−1^ mg^−1^ total protein, respectively (Table 2). These specific activities of GSs were apparent values calculated per mg of total protein in the soluble extracts. The disruption of either GS isoform did not considerably affect the total GS activity, suggesting a compensating increase in the activity of the remaining isoform in each case. Although total GS activity in M2 is similar to that of WT, which is indicative of increased GSI activity, and is consistent with the more intense GSI band on the zymogram for M2, the less intense GSII band for M1 on the zymogram is contradictory to the ^14^C-based GS activity data. The detection of 23% GS activity compared to WT in the nominal “M12” double mutant is in contrast to the lack of detectable bands on the zymogram but likely illustrates the lower sensitivity in the zymogram. Due to the failure of complete segregation, gene expression from the remaining few copies of *sll*1393 would appear to be the source of this GS activity in the nominal “M12”.

The amounts of glycogen produced by WT and GS mutants were determined to further quantify the GS activity of the corresponding isoforms. Glycogen yields of WT, M1, and M2 were 25.3, 23.2, and 22.5 mg g^−1^ wet cell mass, respectively (Table 3). The amount of glycogen production demonstrated that the quantities of glycogen accumulated in both mutants were not substantially reduced. This result is consistent with no decrease in the total GS activity in the individual mutants.

**Table 3:** Effects of *sll*1393 and *sll*0945 deletions on glycogen accumulation and structure, using commercial rabbit-liver glycogen and waxy maize starch for comparison.

Glucan source	Glycogen yield^a^ (mg glycogen g^−1^ wet cell mass)	Average chain-length^b^ (DP_n_)	λmax
Wild type	25±1	9.6±0.4	512
M1 (*sll*1393^−^)	23±2	9±1	490
M2 (*sll*0945^−^)	22.5±0.1	8±1	508
Rabbit-liver glycogen	N/A^c^	11.3±0.0	494
Waxy maize starch	N/A	18.8±0.0	576

a Values given are means ± standard deviation obtained from at least three independent experiments.

b Calculated based on peak area of each chain on HPAEC chromatograms.

*Number*-average degree of polymerization = Σ peak area/Σ (peak area/number of glucose of each chain). Values given are means ± standard deviation obtained from at least three independent experiments.

c Not Applicable.

The activities of AGPase and GBE were also determined in order to explore the effects of the GS mutations on other enzymes of glycogen synthesis (Table 2). Analysis of AGPase activity showed no significant differences among WT, M1 and M2 (*P*<0.05). The AGPase in glycogen production is the rate limiting step [35], and *null*-AGPase in *Synechocystis* sp. PCC6803 by mutation could not produce glycogen structure [36]. Thus, this result suggests that GS mutation does not change the AGPase activity to produce ADP-glucose during glycogen synthesis *in vivo*. However, GBE activity of M2 is significantly higher than those of WT and M1 (*P*<0.05).

### Branch-Structure of Glycogen from M1 was Significantly Different from M2 and WT

**Figure 4:**
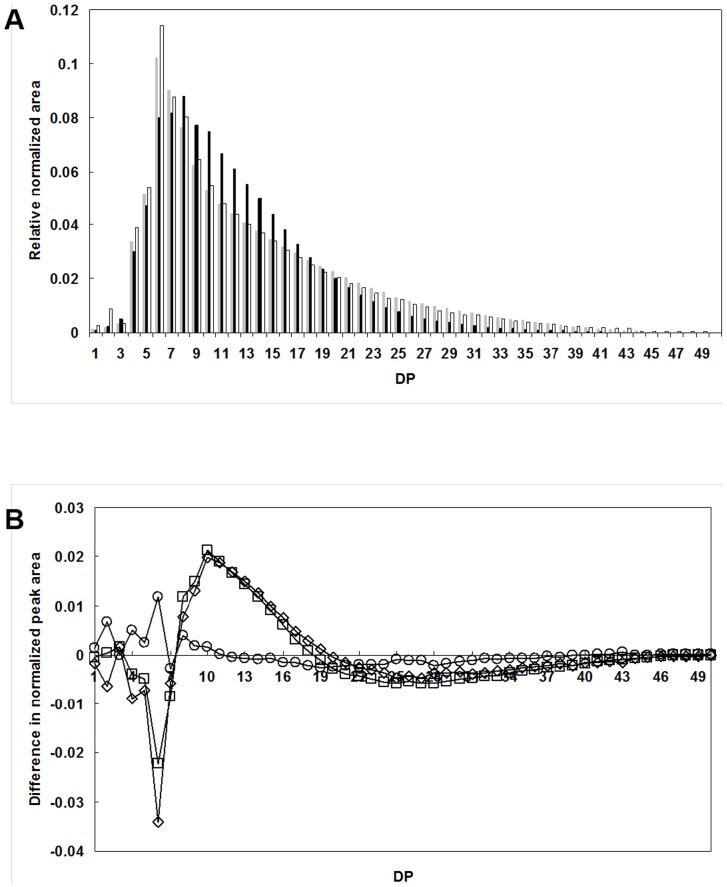
A. Branch chain-length distribution of glycogens from WT (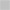), M1 (▪), and M2 (□). Glycogen samples were treated with a debranching enzyme, isoamylase, and the resulting debranched-samples were separated on an HPAEC system. The peak area was calculated and normalized from the chain-profile chromatogram. B. The comparison of difference in the normalized peak area calculated from M1-WT (□), M2-WT (○), or M1-M2 (⋄).

Glycogens produced by the mutants were isolated and their structures were analyzed to identify specific contributions of individual GS isoforms during glycogen synthesis. The branch chain-length distribution of glycogen from M1, M2, and WT was obtained using high-performance anion-exchange chromatography (HPAEC) (Figure 4A). Regardless of strain types, a unimodal distribution of branch chain-length was displayed by isolated glycogen, whereas a bimodal distribution is observed for amylopectin from plant starch [37], [38]. The *number*-average chain-lengths (DP_n_) of glycogens of WT and mutants from *Synechocystis* sp. PCC6803, rabbit-liver and waxy maize starch were determined (Table 3). While DP_n_ did not reflect the chain-length distribution, the DP_n_ of cyanobacterial glycogen samples were similar to various cyanobacterial derivations reported about *Lyngbya-Phormidium-Plectonema* (DP_n_: 11) [39], and *Nostoc muscorum* (DP_n_: 13) [40]. Otherwise, DP_n_ values were slightly shorter than that of rabbit-liver glycogen (DP_n_: 11), but substantially shorter than that of waxy maize starch (DP_n_: 19).

The chain-length distribution of M1-glycogen was quite different from that of WT-glycogen (Fig. 4B). M1-glycogen had fewer short chains (DP 4–7; DP: degree of polymerization) and long chains (>DP 19), but had more intermediate chains (DP 8–18) than did WT. Peaks of chain-lengths of WT, M1, and M2 were at DP 6, 8, and 6, respectively (Fig. 4A). The proportion of each chain was similar to WT in M2 but not in M1 (Figure 4B), except for a larger proportion of short chains (DP 4–11) in M2. Therefore, even though M2 contained an increased level of GBE activity and a loss of GSII activity, the glycogen produced by M2 was fairly similar to that of WT. The relatively minor changes in overall branch-chain length distribution in the absence of GSII suggested that GSI played a predominant role in determining the final structure of glycogen in wild-type *Synechocystis* sp. PCC6803. On the other hand, the preferential biosynthesis of longer chains (≥DP 20) in M2 (GSII^−^), which is unlikely to have resulted from the increased GBE activity, suggests that GSI is capable of extending the glycogen primer in a more progressive way to produce longer chains as predicted based on the darker purple GSI bands in the zymogram. In addition, the great increase of GBE activity in M2 (Table 2) may have affected the glycogen structure. Even if M2 contained twice as much GBE activity as WT, this mutant still produced a much larger proportion of long glucan chains, suggesting that GSI preferably elongated α-glucan chains in a progressive way in spite of the potential interference from the increased GBE activity.

The ratio (%) of β-limit dextrins of WT-, M1-, and M2-glycogens were 48, 40, and 45%, respectively, all of which were larger than that of rabbit-liver glycogen (32%) (Table 4). The higher β-amylolysis value for WT than for M1 indicates shorter exterior chains in M1, which should be a function of the number and length of branch chains. Because the branching enzyme activity, a significant determinant of the number of branches, is similar for both WT and M1, we do not expect a real change in the number of chains. Therefore, the decrease in exterior chain length (ECL) may again be indicative of the specific functions of the lack of GSI in M1. Consistent with this result, the ECL of M1 (5.5) is less than that of WT (6.6). These results suggest that GSI is specifically responsible for the progressive addition of glucose units onto α-glucan chains, thereby forming longer chains. Furthermore, the absence of GSI in M1 may have resulted in fewer chains that have the minimum chain length required to be branched. The lower β-amylolysis value and ECL support the presence of shorter chains in M1, leading to fewer A chains and resulting in a lower A∶B ratio in M1. An explanation for the data obtained for β-amylolysis, ECL and A∶B ratio in M2 in terms of the loss of GSII activity is unclear since the loss of GSII activity is accompanied by the increase in branching enzyme activity. However, the cyanobacterial glycogens showed much less β-amylolysis ratio compared with waxy maize starch (55%). Waxy maize starch displayed much longer ECL (12.3) than any of other glycogens. All glycogens have a very similar molar ratio of A∶B chains between 0.9 and 1.1, whereas waxy maize starch showed a much larger proportion (1.6) of A-chains (Table 4).

**Table 4:** Branch structure of glycogen and starch.

Glucan source	β-Amylolysis (%)^a^	ECL^b^	A∶B ratio^c^
WT	48±2	6.6	1.1∶1.0
M1	40±3	5.5	1.0∶1.0
M2	45±5	5.8	0.9∶1.0
Rabbit-liver glycogen	32±1	5.6	1.1∶1.0
Waxy maize starch	55±1	12.3	1.6∶1.0

a Values given were determined from at least two duplications.

b ECL (exterior chain length) = CL (which is equal to DP_n_ in Table 3)×β-amylolysis (%)+2.0.

c The ratio was calculated based on the mole fraction of (G2+G3) stubs released, by isoamylase and pullulanase hydrolysis, from β-limit dextrin.

Longer exterior chains present in the glycogen of WT and M2 relative to M1 were demonstrated by a longer λmax for the iodine-glycogen complex (Table 3), which increases with increasing chain length. These results agreed with the chain-length distribution analyzed by HPAEC (Figure 4) and a darker purple-color stain shown on the zymogram (Figure 3). M1 has a shorter λmax value (490 nm) for iodine complexes than WT (512 nm), which is consistent with a decrease in the long chains (DP>18) as a result of the absence of GSI activity.

In this study, it is not possible to compare the unique functions of each GS isoform of this cyanobacterium with most of the other bacteria, because many of the other cyanobacteria only contain one GS (mainly GSII type) [31]. Isoforms of GS and soluble starch synthase (SSS) have been reported from a couple of unicellular eukaryotes, *Saccharomyces cerevisiae* [5] and *Chlamydomonas reinhardtii* [41], respectively. The functions of each isoform of SSS have also been studied in plants [42]–[44] with clear evidence that they play distinct roles in amylopectin synthesis. Different patterns of elongation have been reported for various isoforms of starch synthase [16], supporting the conclusion that the different GS isoforms might exhibit different elongation activities.

## Conclusion

**Figure 5:**
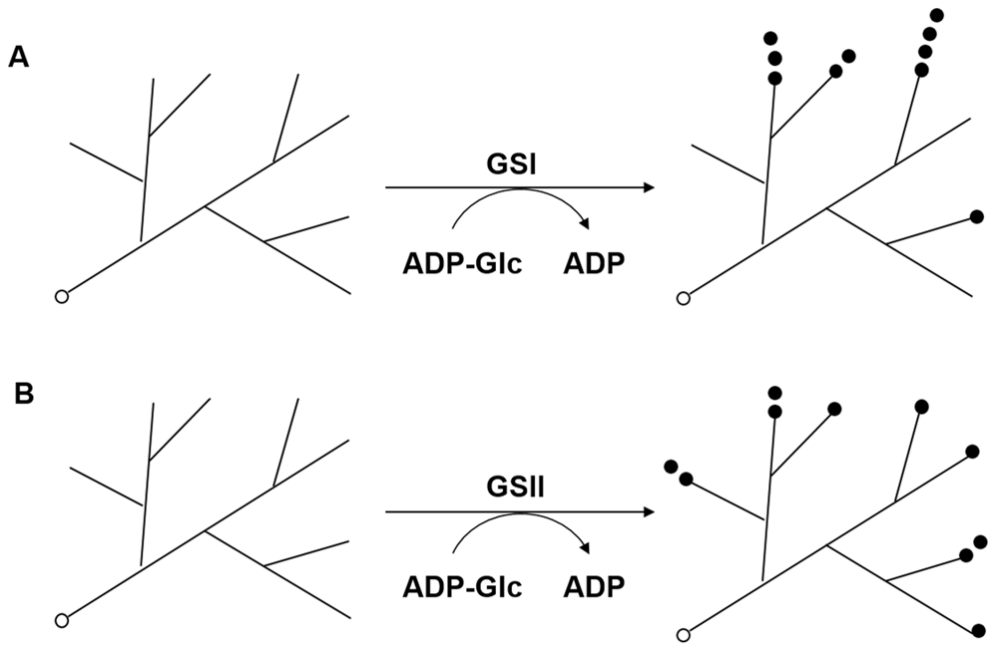
Possible enzyme reactions of the GSI and GSII isoforms in elongation of glycogen primers. Glucose units (•) from ADP-glucose are transferred by GS onto the non-reducing ends of the glycogen primer either progressively by GSI (A) or distributively (B) by GSII. The reducing-end of the glycogen primer is represented by the symbol (○).

The results presented here demonstrate that *Synechocystis* has two GS genes, each of which encodes an active GS protein; however, they have different elongation properties of α-glucosyl units. All research data (the branch chain length distribution, zymogram staining, β-amylolysis and ECL) revealed that the absence of GSI in M1 causes a reduction in longer chains and supports the notion that GSI may preferentially extend chains progressively by adding more glucose units to the same chain, whereas GSII in M2 may add single glucose units distributively one at a time to many chains. Consequently, GSI is apparently involved in generating longer branch chains in the glycogen structure while GSII appears to preferentially form intermediate length chains instead. Different biocatalytic properties of the two GS isoforms were hypothesized as shown in Figure 5. Further studies using purified GS isoforms will be required to confirm the differences in their reaction patterns.
